# DNA polymerase gamma (Polγ) deficiency triggers a selective mTORC2 prosurvival autophagy response via mitochondria-mediated ROS signaling

**DOI:** 10.1038/s41388-018-0404-z

**Published:** 2018-07-23

**Authors:** Sanjit K. Dhar, Vasudevan Bakthavatchalu, Bithika Dhar, Jing Chen, Izumi Tadahide, Haining Zhu, Tianyan Gao, Daret K. St. Clair

**Affiliations:** 1 Department of Toxicology and Cancer Biology, University of Kentucky, Lexington, KY 40536 USA; 2 Division of Comparative Medicine, Massachusetts Institute of Technology, Cambridge, MA 02139 USA; 3 Department of Molecular and Cellular Biochemistry, University of Kentucky, Lexington, KY 40536 USA

## Abstract

Autophagy is a highly regulated evolutionarily conserved metabolic process induced by stress and energy deprivation. Here, we show that DNA polymerase gamma (Polγ) deficiency activates a selective prosurvival autophagic response via mitochondria-mediated reactive oxygen species (ROS) signaling and the mammalian target of rapamycin complex 2 (mTORC2) activities. In keratinocytes, Polγ deficiency causes metabolic adaptation that triggers cytosolic sensing of energy demand for survival. Knockdown of Polγ causes mitochondrial stress, decreases mitochondrial energy production, increases glycolysis, increases the expression of autophagy-associated genes, and enhances AKT phosphorylation and cell proliferation. Deficiency of Polγ preferentially activates mTORC2 formation to increase autophagy and cell proliferation, and knocking down Rictor abrogates these responses. Overexpression of Rictor, but not Raptor, reactivates autophagy in Polγ-deficient cells. Importantly, inhibition of ROS by a mitochondria-selective ROS scavenger abolishes autophagy and cell proliferation. These results identify Rictor as a critical link between mitochondrial stress, ROS, and autophagy. They represent a major shift in our understanding of the prosurvival role of the mTOR complexes and highlight mitochondria-mediated ROS as a prosurvival autophagy regulator during cancer development.

## Introduction

DNA polymerase gamma (Polγ) is a nuclear-encoded, mitochondrially active DNA replication and repair enzyme that is essential for the survival of eukaryotic life [1–5]. Polγ homozygous knockout in mice causes embryonic lethality due to an early developmental defect associated with severe depletion of mitochondrial DNA (mtDNA) [6]. Because mtDNA encodes 13 proteins that, along with over 85 nuclear-encoded proteins, assemble into the oxidative phosphorylation system [7, 8], maintenance of mtDNA levels and integrity is critically important for mitochondrial energy production.

We have previously shown that Polγ becomes nitrated and is subsequently inactivated in UV-induced skin carcinogenesis [9], but the mechanisms by which this occurs are not well characterized. UV irradiation of skin cells triggers the production of nitric oxide, which, when combined with superoxide, forms peroxynitrite (OONO^−^), a very potent oxidant species that modifies the tyrosine residues of proteins. Such modifications are regarded as a marker for nitrative stress [10], and Polγ is highly susceptible to peroxynitrite attack due to the presence of 31 tyrosine residues in its catalytic subunit, including the two highly conserved tyrosines in its active site [11].

The downstream effects of carcinogenic inactivation of Polγ are the object of ongoing investigation. Several lines of evidence have demonstrated that the oxidative stress leading to DNA damage provokes organelle defects which activate autophagic recycling, resulting in either cell death or survival [12]. In the context of many cellular stressors, ranging from hypoxia to DNA damage, autophagy constitutes a key prosurvival response, allowing adaptation to unfavorable conditions [13–15]. Autophagy facilitates the turnover of damaged organelles, including the mitochondria. This process occurs in cancer cells, leading to cell growth and proliferation by elevating glycolysis, which is also known as Warburg effect [16]. Because of the role of Polγ in the maintenance of mtDNA, we propose a link between Polγ activity, mitochondrial integrity, ROS, and autophagy. In this study, we provide evidence that loss of Polγ activity causes mitochondrial stress, leading to metabolic reprogramming, and autophagy via the mammalian target of rapamycin complex 2 (mTORC2).

## Results

### Nitration of Polγ and its effect on enzymatic activity

**Fig. 1:**
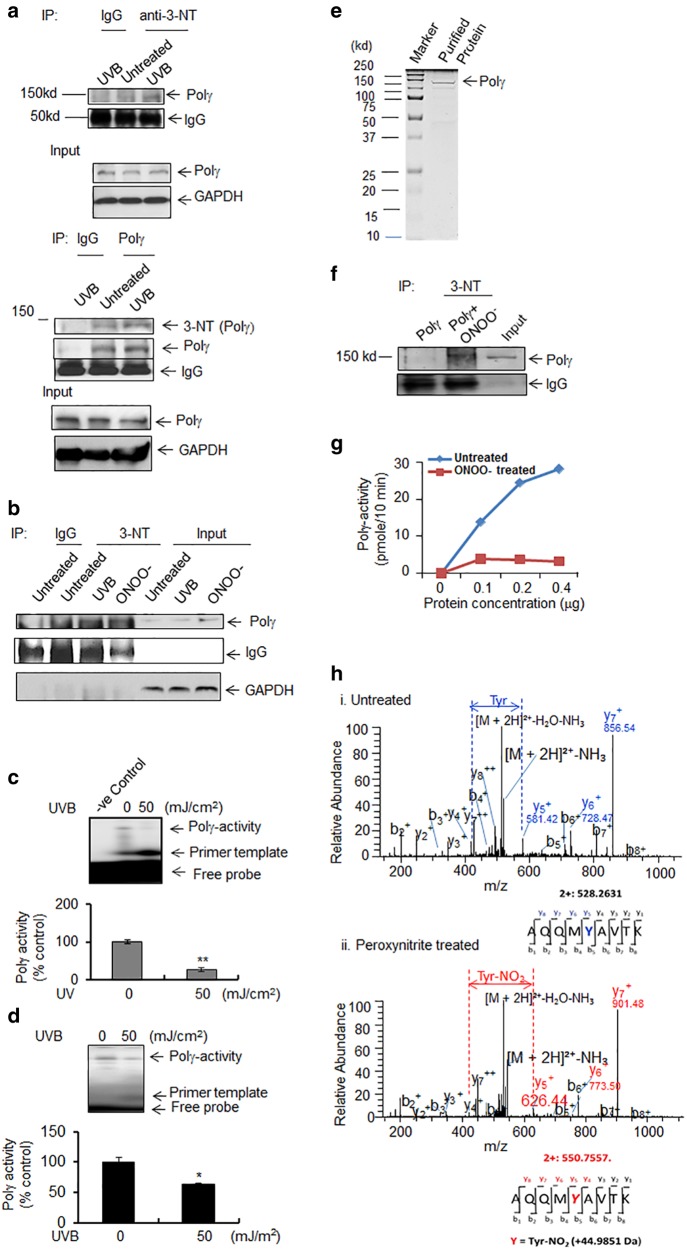
Polγ nitration and activity. **a** Detection of Polγ nitration after UVB irradiation (50 mJ/cm^2^ × 1 h) in human primary epidermal keratinocytes using 3-nitrotyrosine immunoprecipitation followed by western blotting with Polγ antibody or Polγ antibody-mediated immunoprecipitation followed by western blotting with 3-nitotyrosine antibody. Both IgG and inputs were provided as loading control. **b** Detection of Polγ nitration by immunoprecipitation after UVB treatment (50 mJ/cm^2^) in JB6 cells using 3-nitrotyrosine antibody with exposure to authentic peroxynitrite (ONOO^−^) as a positive control. Both IgG and inputs were provided as loading control. **c** Polγ activity in human keratinocytes with or without UVB treatment was detected by 20% acrylamide/7 M urea gel electrophoresis and autoradiography following primer extension using mtDNA-specific primers (see Materials and methods). **d** Polγ activity in JB6 cells was detected as described in panel (**c**). **e** SDS-PAGE of purified recombinant mouse Polγ protein and visualization by Coomassie brilliant blue staining. **f** Purified Polγ proteins were immunoprecipitated with 3-nitrotyrosine antibody before and after treatment with peroxynitrite (250 μM). Polγ proteins were detected by western blotting using an anti-Polγ antibody. Both IgG and inputs were used as loading control. **g** Activity of purified Polγ protein was measured by dTTP incorporation in the presence or absence of peroxynitrite as described in the Materials and methods. **h** Nitration of tyrosine residues was detected by mass spectrometry. Each peak in the MS/MS spectrum represents relative abundance of peptide fragment and the tyrosine residues of the peptide fragment are shown under each graph. For all quantifications (**c**, **d**), each data point represents the mean ± SD of three individual samples. For all panels, representative figures from three repeated experiments are shown. Statistical analysis was performed using *t* tests. Statistical significance is indicated by asterisks: *p < 0.05 and **p < 0.01

It has been shown that UVB increases peroxynitrite generation [17, 18]. To elucidate whether and how UVB treatment causes Polγ nitration, we exposed primary human epidermal keratinocytes or JB6 cells to UVB radiation and used a 3-nitrotyrosine antibody to detect nitrated Polγ. The nitration of Polγ was detected in both primary human epidermal keratinocytes and JB6 cells following UVB radiation (Fig. 1a, b). Further, reverse immunoprecipitation was performed using Polγ antibody and the nitration of Polγ was confirmed by western blotting using 3-nitrotyrosine antibody after UVB treatment (Fig. 1a bottom panel). To verify the nitration-mediated inactivation of the enzymatic activity upon UVB treatment, we measured Polγ activity using isolated mitochondria. Our data show that Polγ activity in human and murine keratinocytes is significantly decreased following UVB treatment (Fig. 1c, d). These results support our previous findings and confirm that Polγ becomes nitrated after UVB irradiation in human and murine keratinocytes and consequently loses enzymatic activity.

### Identification of nitration site in Polγ

To precisely identify the nitrated amino acid residue(s) in Polγ, we purified full-length recombinant mouse Polγ (Fig. 1e) and exposed it to authentic peroxynitrite in vitro. The nitration of Polγ by peroxynitrite was confirmed by immunoprecipitation using a 3-nitrotyrosine antibody followed by western blotting against Polγ (Fig. 1f). A Polγ reverse transcriptase activity assay was performed using purified protein following peroxynitrite treatment. The activity of peroxynitrite-treated Polγ was significantly reduced compared to the saline-treated protein (Fig. 1g).

To identify the specific nitrotyrosine modification of Polγ, we digested the purified peroxynitrite-treated Polγ protein with trypsin and analyzed the peptide mixture by mass spectrometry. Nitrated peptides were identified using liquid chromatography electrospray ionization (LCESI)-MS/MS analysis. It has been demonstrated that introduction of a nitro (NO_2_) group onto a tyrosine results in a difference in m/z ratio of +45 [19]. Figure 1h shows the LCESI-MS/MS spectra of the tryptic peptides of untreated Polγ and peroxynitrite-treated Polγ. In the MS/MS spectra, the tyrosine contained in peptide y5 has an m/z of 581.42 in the untreated sample, whereas peroxynitrite-treated peptide y5 has an m/z ratio of 626.44. This difference in molecular mass indicates the nitration of a single tyrosine residue in Polγ, and the MS/MS spectra identified this peptide as containing tyrosine 964 in the catalytic domain of the protein. Interestingly, Y964 is well conserved in a variety of species ranging from humans to *Saccharomyces* (Supplementary Figure S1).

### Polγ deficiency causes mitochondrial DNA damage and mitochondrial dysfunction

**Fig. 2:**
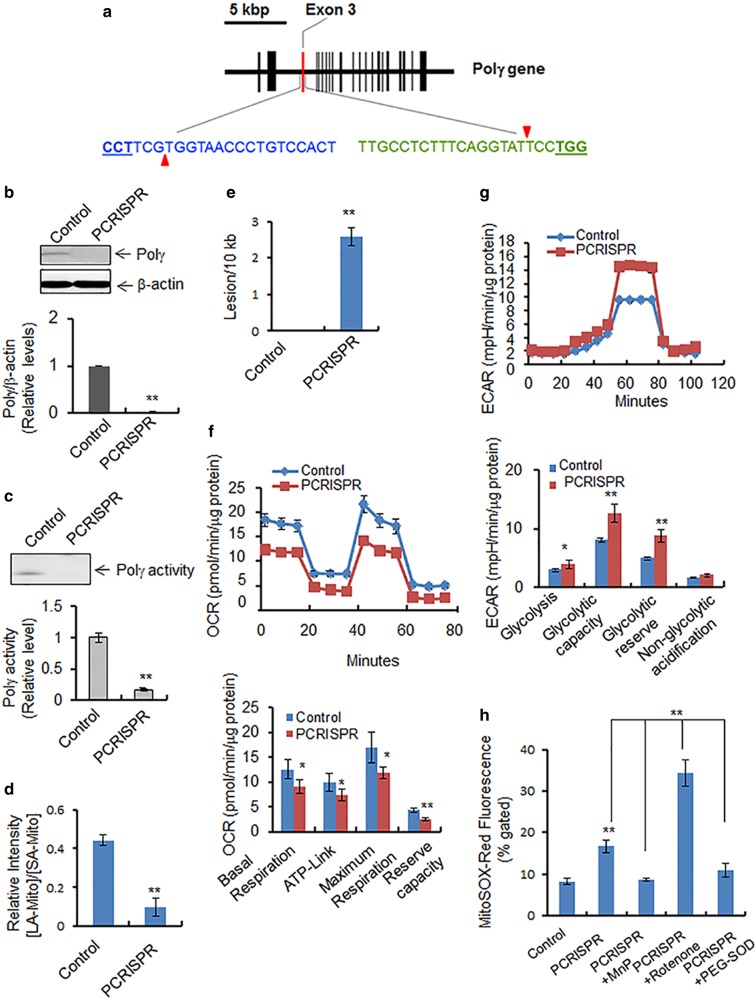
Effects of Polγ suppression on mitochondria. **a** The mouse Polγ gene structure in chromosome 7 is shown schematically. The vertical bars indicate the exons. The exon 3 targeted by CRISPR is highlighted in red. Two 20-nucleotide guide sequences of CRISPR are shown, in blue and in green. The pam sequences are underlined, and red triangles indicate the incision sites by Cas9n. **b** Polγ protein levels were measured by western blotting in PCRISPR JB6 clones. **c** Polγ activity in mitochondria isolated from PCRISPR cells was detected by primer extension followed by autoradiography (see Materials and methods). **d** The PCR product ratio of long-amplicon (LA) mtDNA to short-amplicon (SA) mtDNA in control and Polγ-deficient PCRISPR cells which is normalized by mitochondrial mass. **e** The number of DNA lesions per 10 kb mtDNA in Polγ-deficient PCRISPR cells following normalization with mitochondrial mass. Note that the mitochondrial masses of control and PCRISPR cells were obtained by staining the cells with NAO, a mitochondria-specific dye, followed by flow cytometry. **f** Mitochondrial oxygen consumption was measured as described in Materials and Methods. OCR oxygen consumption rate. **g** Glycolysis was measured as described in Materials and Methods. ECAR extracellular acidification rate. **h** The level of mitochondrial reactive oxygen was measured by quantifying the MitoSox Red fluorescence in Polγ-deficient PCRISPR cells in the presence and absence of MnP (MnTnBuOE-2-PyP^5+^), with positive and negative controls. The mean fluorescence intensity of MitoSox Red was determined using flow cytometry. The concentration of cellular superoxide was estimated by quantification of fluorescence intensity. Rotenone was used as a positive control for generation of ROS. PEG-SOD (Superoxide dismutase–polyethylene glycol from bovine erythrocytes) was also used as a control to remove superoxide generated by MitoSox Red. The fluorescence intensity of MitoSox Red is normalized by total mitochondrial mass. In all bar graphs and line graphs, each data point represents the mean ± SD of three individual samples. Each experiment was repeated at least three times and statistical analysis was performed using *t* tests for two groups or one-way ANOVA analysis and Bonferroni’s post-test for multiple-group comparisons. Statistical significance is indicated by asterisks: *p < 0.05 and **p < 0.01

Polγ plays a pivotal role in protecting the mitochondrial genome from oxidative damage [20]. To demonstrate this role of Polγ, we generated stable Polγ-knockout cell lines using CRISPR-mediated gene editing technology (Fig. 2a). Polγ-knockout PCRISPR cells were selected by using puromycin selection antibiotics. Among several stable positive clones, complete knockdown PCRISPR clones were selected and propagated. To rule out the off-target effect of the CRISPR method, we assessed the mitochondrial resident MnSOD proteins by western blotting. Furthermore, we detected Polγ in PCRISPR cells by overexpressing wild-type and mutant Polγ proteins (Figure S5). The expression of Polγ was determined by western blotting and the activity by primer extension using isolated mitochondria from Polγ-knockout (PCRISPR) cells. As shown in Fig. 2b, c, both Polγ protein levels and Polγ activity were diminished in PCRISPR cells.

To investigate the impact of Polγ deficiency on mtDNA damage, we amplified a 6.9 kb DNA fragment and 0.2 kb DNA fragment (long and short amplicons, respectively) using mitochondrial gene-specific primers. The amplified mtDNA was quantified and normalized against nuclear DNA to assess the relative changes in the mitochondrial amplicons. Further, we assessed the relative levels of DNA damage relative to the levels of mitochondrial mass [21]. To assess the mitochondrial mass, we stained both control and PCRISPR cells using mitochondria-specific cardiolipin binding dye NAO (10-nonylacridine orange bromide) followed by flow cytometry. The NAO uptake in control and PCRISPR was used to normalize the mitochondrial DNA damage in control and PCRISPR cells, respectively. The data from PCR analysis shown in Fig. 2d demonstrate that the amount of long-amplicon mtDNA relative to short-amplicon mtDNA is significantly lower in Polγ-deficient CRISPR cells as compared to control cells. The observed number of mtDNA lesions per 10 kb was greater in Polγ-deficient CRISPR cells than in control cells, indicating an increase in mtDNA damage (Fig. 2e).

To determine how Polγ deficiency affects mitochondrial function, we measured cellular bioenergetics. The data presented in Fig. 2f show that oxygen consumption was decreased in PCRISPR cells compared to controls, resulting in a decrease in basal respiration, ATP-linked respiration, maximum capacity, and reserve respiratory capacity. Conversely, the extracellular acidification rate was increased in PCRISPR cells as compared to controls, resulting in increases in glycolysis, glycolytic capacity, and glycolytic reserve (Fig. 2g). PCRISPR cells also exhibited an increase in lactate levels compared to control cells (Supplementary Figure S2). These results suggest that the deficiency of Polγ in PCRISPR cells causes an increase in mitochondrial stress, which leads to the impairment of oxidative phosphorylation and a shift toward glycolysis.

Mitochondrial ATP production is driven by electrons being passed along the electron transport chain (ETC), with a considerable number leaking as superoxide radicals/anions (O_2_^•−^) during the process. To determine if Polγ deficiency contributes to the generation of O_2_^•–^ in mitochondria, we used fluorometric analysis, with positive and negative controls, to quantify O_2_^•−^ in Polγ-deficient PCRISPR and control JB6 cells. Mitochondrial superoxide anion was measured using MitoSox Red, a fluorogenic dye that specifically targets mitochondria in live cells. The relative mitochondrial mass was used to normalize MitoSox Red to quantify mitochondrial superoxide anion, with rotenone as a positive control. The levels of superoxide radicals in Polγ-deficient PCRISPR cells were significantly higher than the levels in control cells (Fig. 2h). Rotenone, an inhibitor of ETC complex I, is able to inhibit mitochondrial oxidative phosphorylation, leading to generation of high levels of superoxide radicals. The results demonstrate a higher level of O_2_^•−^ in Polγ-deficient cells exposed to rotenone (Fig. 2h).

### Deficiency of Polγ activates autophagy

As autophagy is critical for old and damaged organelles, we investigated the role of Polγ in autophagy. Microtubule-associated protein light chain 3 (LC3) is an abundant cytoplasmic protein that is cleaved and lipidated during initiation of autophagy (forming LC3B or LC3 II) and is incorporated into autophagosomal membranes in a punctate pattern [22]. LC3B is a downstream indicator of the autophagic pathway and participates in autophagosome formation and maturation [23].

**Fig. 3:**
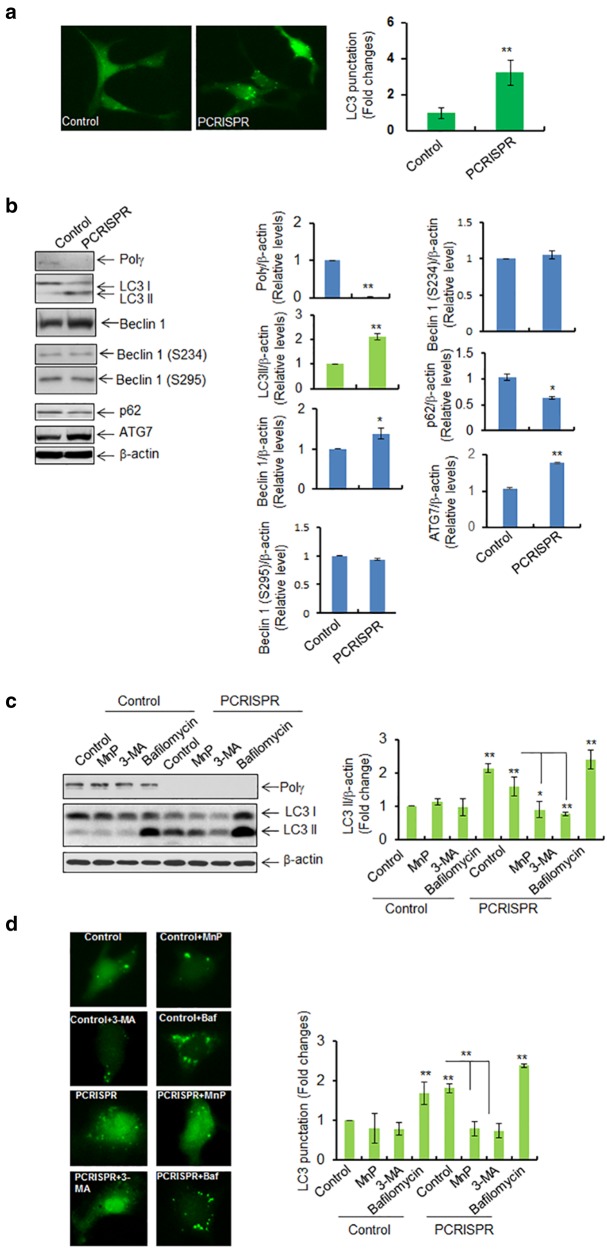
Suppression of Polγ increases the autophagic response. **a** LC3 punctation was detected in Polγ-deficient PCRISPR cells following expression of GFP-LC3. For each cell type, 100 GFP-positive cells were counted, and the right panel shows the quantification of punctated cells. **b** Western blot analysis and quantification of the increase in LC3 II, beclin 1, phosphorylated beclin 1, ATG7, and the decrease in p62 in Polγ-deficient PCRISPR cells compared to controls. **c** Autophagy flux was detected by western blotting in PCRISPR cells following treatment with autophagy inhibitors (MnP; MnTnBuOE-2-PyP^5+^ 3-MA; 3-methyle adenine and Bafilomycin). The bar graph shows the quantification of LC3 II band intensity normalized to β-actin. **d** LC3 punctation was detected using fluorescence microscopy with or without autophagy inhibitors. The bar graph shows the quantification of punctated cells (100 GFP-positive cells were counted for each cell type). In all panels, each experiment was repeated at least three times. In the bar graphs, each data point represents the mean ± SD of three individual samples. Statistical analysis was performed using *t* tests for two groups or one-way ANOVA analysis and Bonferroni’s post-test for multiple-group comparisons. Statistical significance is indicated by asterisks: *p < 0.05 and **p < 0.01

To test the role of Polγ in autophagy, LC3-GFP expression vectors were transfected into Polγ-deficient CRISPR cells. We then determined the levels of LC3 punctation and LC3 II formation by fluorescence microscopy and western blotting, respectively. Figure 3a shows that LC3 punctation was significantly increased in PCRISPR cells as compared to control cells. Consistent with this finding, the endogenous level of LC3 II formation was increased in PCRISPR cells (Fig. 3b).

We further assessed the critical upstream autophagy regulators beclin 1 and Atg7. Beclin 1 is responsible for initiation of autophagosome formation [24, 25]. Consistent with the LC3 II formation, beclin 1 was significantly increased in PCRISPR cells as compared to control cells (Fig. 3b). The autophagic function of Beclin 1 is regulated by its phosphorylation at S234 and S295 [26]. The Beclin 1 phosphorylation was not altered in PCRISPR cells as compared to control (Fig. 3b). The autophagy initiator ATG7 was also significantly increased in PCRISPR cells as compared to controls (Fig. 3b). Consistent with an increase in autophagy, the level of SQSTM1 (p62) protein, which recognizes toxic cellular waste, was reduced in PCRISPR cells.

Autophagy flux was assessed to confirm the increase of autophagy in Polγ-deficient CRISPR cells as detected by western blotting and fluorescence microscopy (Fig. 3c, d). The results show that higher levels of LC3 II in PCRISPR cells, as compared to control cells, can be suppressed by pretreatment with the ROS inhibitor MnP or the PI3k inhibitor 3-MA. On the other hand, Polγ-deficiency-mediated increases of LC3 II levels and LC3 punctation were further increased after treatment with bafilomycin for 24 h as compared to PCRISPR alone. These results confirm that a deficiency of Polγ increases autophagy.

### Mutation of Y964 inactivates Polγ and triggers autophagy

**Fig. 4:**
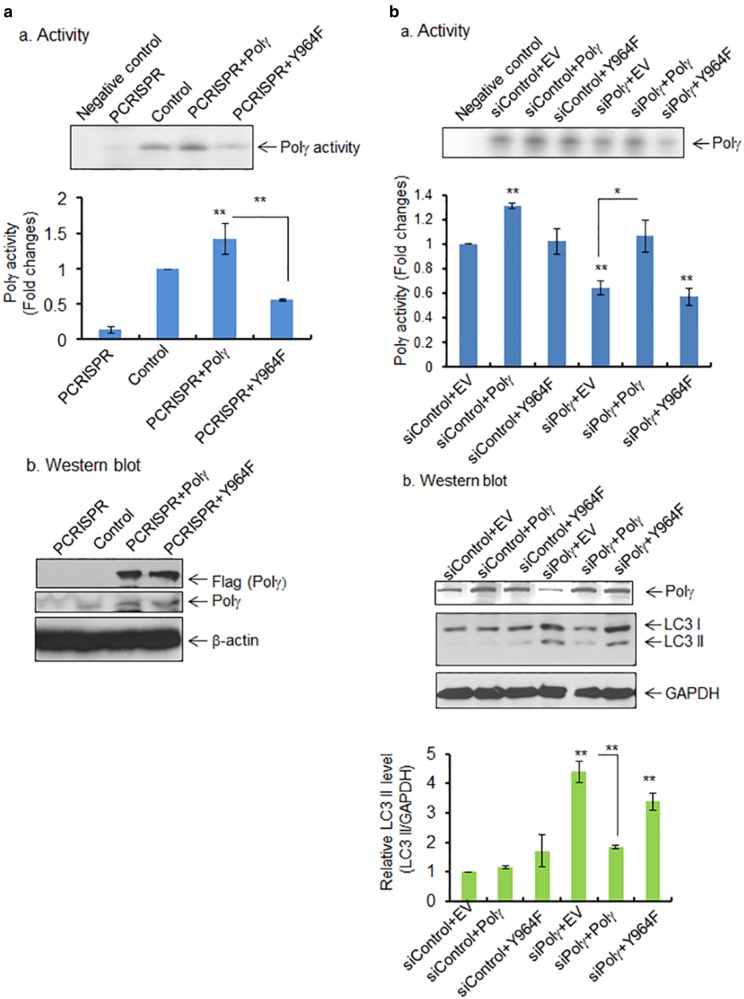
Effects of tyrosine mutation on Polγ activity and autophagy. **a**, panel a The activity of Polγ in mitochondria following transfection of wild-type and mutant (Y964F) Polγ expression vectors in Polγ-deficient PCRISPR cells. Relative activity of Polγ was measured and expressed as the fold change (bottom panel). The specificity of Polγ activity was validated by comparing with negative control, which contains template−primer mixture and radio-labeled ATP without enzyme. Panel b The ectopic expression of wild-type and mutant Polγ was detected by western blotting using Flag antibody. Endogenous level of Polγ in PCRISPR cells is also shown. **b**, panel a The Polγ activity in JB6 cells following coexpression of wild-type or mutant (Y964F) Polγ plasmid along with Polγ siRNA. Relative activity of Polγ was measured and expressed as the fold change (bottom panel). Panel b The suppression of Polγ by siRNA and the overexpression of wild-type and mutant Polγ were confirmed by western blotting using Polγ antibody. The relative level of LC3 II formation in JB6 cells following coexpression of wild-type or mutant (Y964F) Polγ plasmid along with Polγ siRNA or control siRNA is shown (bottom panel). The specificity of Polγ activity was validated by comparing with negative control, which contains template−primer mixture and radio-labeled ATP without enzyme. Each data point represents the mean ± SD of three individual samples. Statistical analysis was performed using one-way ANOVA analysis and Bonferroni’s post-test for multiple-group comparisons. Statistical significance is indicated by asterisks: *p < 0.05 and **p < 0.01

As shown in Fig. 1h, using mass spectrometric analysis, we identified the tyrosine nitration at the Y964 site in the catalytic domain of Polγ. To examine the significance of tyrosine nitration in Polγ, we performed site-directed mutagenesis to generate a Polγ-Y964F mutant. We then overexpressed wild-type Polγ or the Y964F mutant in Polγ-deficient CRISPR cells as well as Polγ-knockdown cells and compared the reverse transcriptase activity of Polγ. Equal overexpression of both wild-type and mutant proteins following transfection were confirmed by western blotting (Fig. 4a, b). Overexpression of Polγ in PCRISPR cells significantly increased the enzymatic activity as compared with PCRISPR controls (Fig. 4a). Overexpression of mutant Polγ increased the Polγ activity about half as much as overexpression of the wild-type protein, suggesting that the Y964F mutation causes a loss of Polγ activity (Fig. 4a). Similar results were observed in JB6 cells with knockdown of endogenous Polγ by siRNA (Fig. 4b).

Next, we determined how the expression of wild-type and mutant Polγ affects the level of LC3 II formation observed in Polγ-deficient cells. Consistent with the results for Polγ activity, overexpression of wild-type Polγ suppressed the LC3 II formation which was enhanced in Polγ-deficient cells (Fig. 4c). However, overexpression of mutant Polγ was not able to suppress autophagic LC3 II formation (Fig. 4c). These results show that the mutation of Y964 in the catalytic domain significantly suppresses the enzymatic activity of Polγ and consequently limits its ability to prevent autophagy.

To examine the biological relevance of Polγ deficiency, we overexpressed wild-type and mutant Polγ in JB6 cells and assessed LC3 II formation after UVB treatment. The autophagic LC3 II formation was significantly increased upon UVB radiation and was attenuated by wild-type Polγ overexpression but not mutant Polγ overexpression (Figure S4).

### Deficiency of Polγ activates mTOR/AKT pathways

**Fig. 5:**
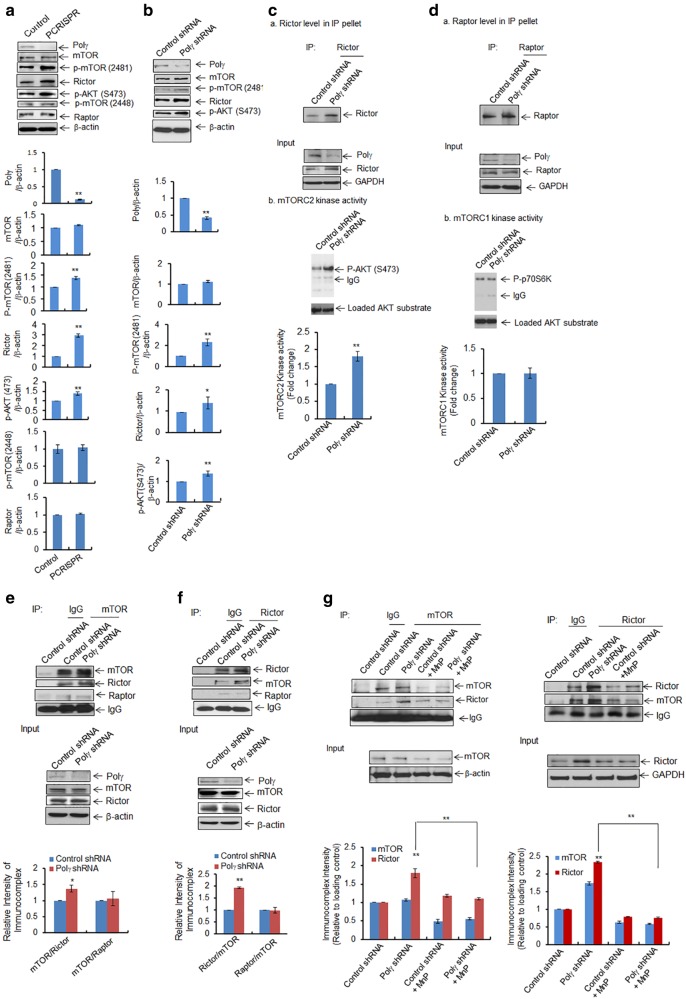
Activation of AKT/mTOR pathway following Polγ deficiency. **a**, **b** Increased levels of mTOR phosphorylation at S2481, AKT phosphorylation at S473, and Rictor were detected by western blotting in **a** Polγ-deficient PCRISPR cells and **b** cells stably expressing Polγ shRNA. **c**, **d** In vitro mTOR kinase activities were measured by using specific substrates for mTOR complexes coupled with immunoprecipitation and western blotting in cells stably expressing Polγ shRNA. **c** For mTORC2 kinase activity, total cell lysates were immunoprecipitated with Rictor antibody. Panel a The immunoprecipitated proteins were detected by western blotting using Rictor antibody. Panel b Immunoprecipitated product was then incubated with purified AKT. The phosphorylated AKT (S473) was detected as an indicator of mTORC2 kinase activity. **d**, Panel a The immunoprecipitated proteins were detected by western blotting using Raptor antibody. For mTORC1 kinase activity, total cell lysates were immunoprecipitated with raptor antibody. Immunoprecipitated product was then incubated with purified p70S6K protein and the phosphorylated p70S6K was detected by western blotting as an indicator of mTORC1 activity. **e** Coimmunoprecipitation of Rictor and Raptor with mTOR from the total cell lysates of cells expressing Polγ shRNA. Input controls are provided as loading control. **f** Reverse coimmunoprecipitation with Rictor antibody followed by western blotting performed with Rictor, mTOR, and Raptor antibodies. Input controls were provided as loading control. **g** Immunoprecipitation was also carried out using mTOR antibody in JB6 cells stably expressing Polγ shRNA following pretreatment with MnP (20 μM × 24 h). The immunoprecipitated products were analyzed by western blotting using antibodies against mTOR and Rictor. Input controls were added as loading control. Similarly, reverse immunoprecipitation was performed using Rictor antibody in Polγ-deficient cells following pretreatment with MnP. Input controls are also shown as loading control. All immunoprecipitated proteins were normalized with house-keeping loading control and quantified. Each experiment was repeated at least three times. In the bar graphs, each data point represents the mean ± SD of three individual samples. Statistical analysis was performed using *t* tests for two groups or one-way ANOVA analysis and Bonferroni’s post-test for multiple-group comparisons. Statistical significance is indicated by asterisks: *p < 0.05 and **p < 0.01

How Polγ deficiency leads to autophagy is not clear. We hypothesized that a deficiency of Polγ that causes aberrant mitochondrial function could activate stress signals that affect the mTOR signaling pathway. To test this hypothesis, we investigated mTOR activation in both PCRISPR cells and stable Polγ-knockdown cells. As shown in Fig. 5, the phosphorylation of mTOR (2481) was significantly increased in Polγ-deficient CRISPR cells (Fig. 5a) as well as Polγ-knockdown cells (Fig. 5b) as compared to the respective controls. Consistent with mTOR phosphorylation, AKT phosphorylation was also increased significantly. A remarkable increase in Rictor, which is a component of mTORC2, was observed in PCRISPR as well as in Polγ-knockdown cells. However, no change in level occurred in Raptor, a component of mTORC1, suggesting that mTORC2 has the predominant role in autophagy mediated by Polγ deficiency. To demonstrate mTORC2 activity, we employed an in vitro kinase assay using purified recombinant human AKT as the substrate. mTORC2, which was immunopurified using a Rictor affinity column, was able to phosphorylate AKT (S473), and the levels of phosphorylation of AKT were increased in Polγ-deficient cells as compared to control cells (Fig. 5c). Similarly, to measure mTORC1, we performed an in vitro kinase assay using purified recombinant S6K as the substrate. mTORC1 was immunoprecipitated with Raptor antibody followed by addition of S6K. As shown in Fig. 5d, the phosphorylated p70S6K did not change in Polγ-deficient cells as compared to the corresponding control cells. Immunopurified Rictor or Raptor from affinity column was confirmed by western blotting with Rictor and Raptor antibody, respectively (Fig. 5c, panel a, d, panel a). Equal loading of substrate was assured by detecting substrate levels using immunoblotting with AKT or p70S6K antibody, respectively (Fig. 5c, d). These results demonstrate the selective increase of mTORC2 in Polγ-deficient cells. Next, we investigated mTOR complex formation in stable Polγ-knockdown cells using immunoprecipitation. The co-immunoprecipitation data show that mTOR pulled down more Rictor than Raptor (Fig. 5e). Reverse co-immunoprecipitation data show that Rictor pulled down more mTOR in Polγs, suggesting the preferential formation of mTORC2 following Polγ suppression (Fig. 5f).

To address whether mTORC2 that is preferentially activated in Polγ-deficient cells is sensitive to an increase in ROS, we used ROS scavenger manganese superoxide dismutase mimetics (MnP) and then coimmunoprecipitated with mTOR antibody. The results (Fig. 5g) show that more Rictor was pulled down with mTOR antibody in Polγ-deficient cells as compared to controls. Treatment of Polγ-deficient cells with MnP significantly reduced the Rictor levels in the immunocomplex, suggesting that mTOR/Rictor complex formation is ROS-dependent. Consistently, reverse immunoprecipitation with Rictor antibody in Polγ knockdown cells showed a decrease of mTOR−Rictor interaction after MnP treatment (Fig. 5g). In addition, MnP treatment decreased the Rictor protein level and AKT phosphorylation suggesting that oxidative stress induces AKT activation (Figure S3B). Further, inhibition of AKT phosphorylation was achieved by treating the cells with the AKT inhibitor as well as by siRNA silencing, which significantly suppressed the Polγ-deficiency-mediated LC3 II levels, suggesting that the AKT pathway is associated with oxidative stress-induced survival autophagy (Supplementary Figure S3A, S3C).

### Polγ deficiency-mediated autophagy is Rictor-dependent

**Fig. 6:**
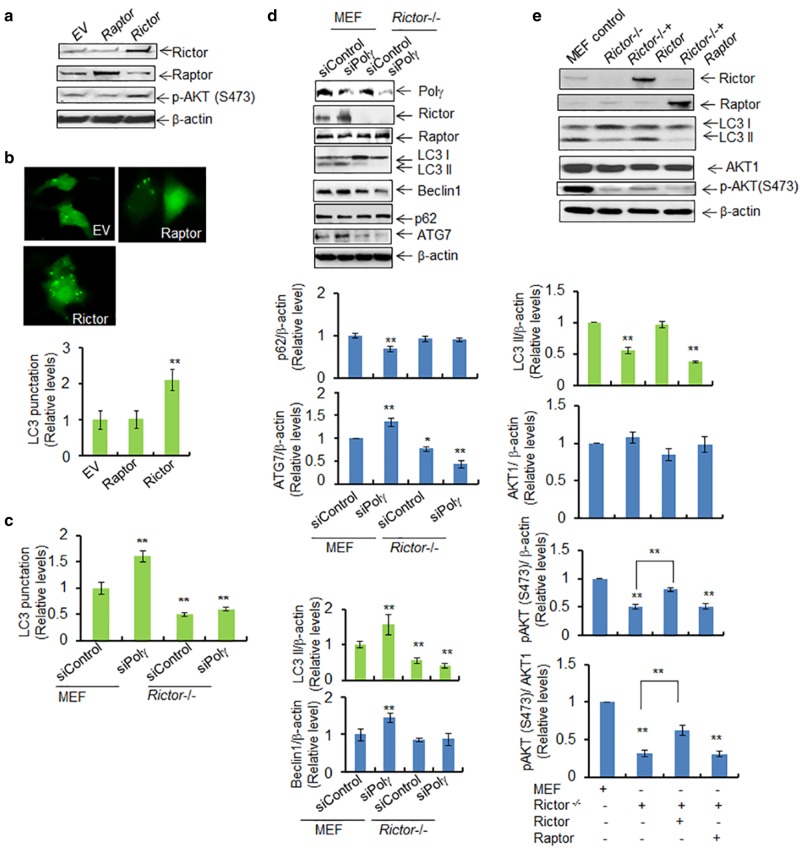
Polγ deficiency-mediated autophagy is RICTOR-dependent. **a** Western blotting shows the overexpression of Rictor or Raptor following transfection of the corresponding vectors in JB6 cells. AKT phosphorylation at Ser473 in the presence of the overexpressed Rictor was also detected by western blotting. Plasmid vector without any protein expression sequence was used as control and is designated by EV. **b** Detection of LC3 punctation following overexpression of Rictor or Raptor. Quantification of LC3 punctation in Raptor- and Rictor-overexpressing JB6 cells is shown (bottom). **c** Quantification of LC3 punctation in wild-type MEF and Rictor-knockout MEF cells following Polγ siRNA transfection. **d** Effects of Polγ deficiency in MEF and Rictor-knockout cells are achieved by expression of Polγ siRNA. The endogenous autophagy markers (LC3 II, beclin 1, ATG7, and p62) were detected by western blotting. The right panels show the corresponding quantification. **e** Overexpression of Rictor or Raptor in Rictor-knockout MEF cells and the level of LC3 II were determined by western blotting. AKT phosphorylation at Ser473 induced by Rictor overexpression was also detected by western blotting. In all panels, each experiment was repeated at least three times, and statistical analysis was performed using one-way ANOVA analysis followed by Bonferroni’s post-test for multiple-group comparisons. Statistical significance is indicated by asterisks: *p < 0.05 and **p < 0.01

The role of mTORC2 in autophagy is not well understood. We thus tested the role of Rictor vs. Raptor in autophagy by employing Rictor suppression or overexpression. We first confirmed by western blotting that the proteins were ectopically expressed (Fig. 6a). Our data show that the basal level of LC3 punctation is significantly increased following overexpression of Rictor but not of Raptor in mouse skin cells (Fig. 6b). To further demonstrate the role of Rictor in Polγ deficiency-mediated autophagy, we suppressed Polγ using siRNA in stable Rictor-knockout MEF cells. Rictor-knockout reversed the increase in LC3 punctation observed in MEF cells with overexpression of Polγ siRNA (Fig. 6c). In addition, as shown in Fig. 6d, Rictor-knockout cells have decreased levels of LC3 II, beclin 1, and ATG7, but not p62, as compared to control MEF cells. Suppression of Polγ by siRNA in MEF cells resulted in increased LC3 II, beclin 1, and ATG7 expression. By contrast, suppression of Polγ by siRNA in Rictor-knockout MEF cells did not increase LC3 II, beclin 1, or ATG7 expression (Fig. 6d). These results suggest that Rictor plays an important role in Polγ deficiency-mediated autophagy.

To further demonstrate the role of Rictor, we modulated Rictor levels by overexpression. As shown in Fig. 6e, we observed an increase in LC3 cleavage to LC3 II following overexpression of Rictor but not Raptor in Rictor-knockout MEF cells. Consistently, AKT phosphorylation was increased in Rictor-knockout MEF cells following overexpression of Rictor but not Raptor. These results are consistent with the idea that Rictor plays a critical role in inducing autophagy in response to Polγ deficiency.

### Deficiency of Polγ enhances cell proliferation

**Fig. 7:**
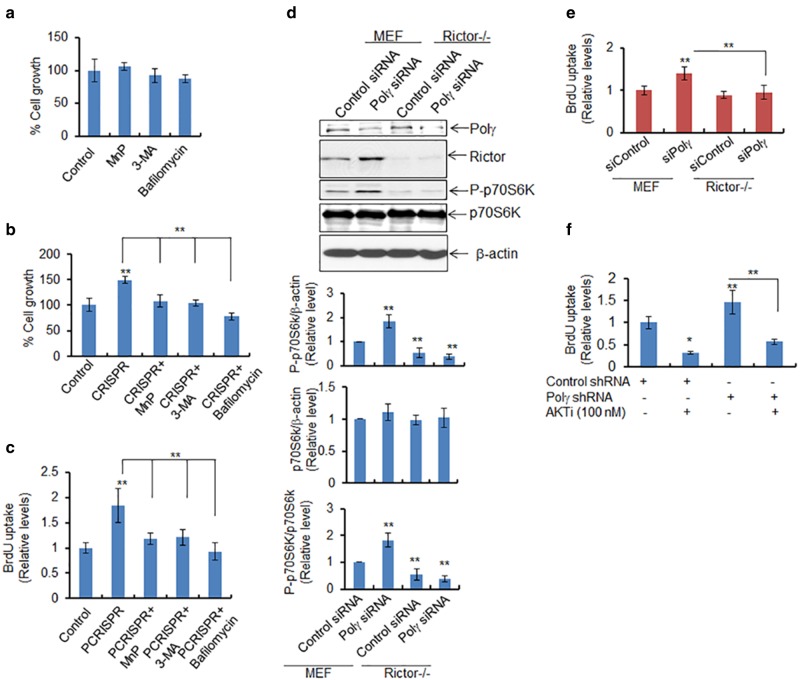
Deficiency of Polγ enhances autophagy-dependent cell proliferation. **a** The cytotoxicity of ROS and autophagy inhibitors (MnP, 20 μM; 3-MA, 2.5 mM; and Bafilomycin, 50 nM) was assessed in JB6 cells 24 h after treatment by determining cell growth. **b** Cell growth was also measured in PCRISPR cells following treatment with the indicated inhibitor. **c** BrdU uptake was determined as a measure of cell proliferation in PCRISPR cells with or without ROS or autophagy inhibitors. **d** Phosphorylation of p70S6K was detected by western blotting in wild-type and Rictor-knockout MEF cells following suppression of Polγ by siRNA. **e** Cell proliferation was determined in Rictor-knockout cells after Polγ suppression using the BrdU uptake assay (assay details are given in Materials and Methods section). **f** Cell proliferation was also determined in Polγ-knockdown cells following treatment with AKT inhibitor using the BrdU uptake assay. In all panels, each data point represents the mean ± SD of three individual samples. Statistical analysis was performed using one-way ANOVA analysis and Bonferroni’s post-test for multiple-group comparisons. Statistical significance is indicated by asterisks: *p < 0.05 and **p < 0.01

We then asked whether Polγ deficiency enhances cell proliferation, because autophagy can function in both cell survival and cell death pathways. We employed a gold standard soft-agar colony formation assay to assess the cell proliferation. Our data show that in treated cells vs. control cells, MnP, 3-MA, and bafilomycin had no effect on cell growth (Fig. 7a). Cell growth was significantly increased in PCRISPR cells as compared to controls (Fig. 7b), and the increased cell growth was suppressed by inhibitors of autophagy in Polγ-deficient cells. The DNA synthesis-based cell proliferation assay using BrdU uptake was consistent with the increase in cell growth (Fig. 7c). These results suggest that a deficiency of Polγ increases cell proliferation.

To address whether Rictor has a direct role in cell proliferation, we investigated the effects of Rictor in the cell proliferation assay. The increase in phosphorylated p70S6K (Th389) was also inhibited in Rictor-deficient Polγ-knockdown cells (Fig. 7d). Figure 7e shows that the increase in BrdU uptake in Polγ-deficient cells was abolished in Rictor-deficient cells. Consistently, BrdU uptake was increased in stable Polγ-knockdown cells, and was significantly reduced again upon treatment with AKT inhibitor (Fig. 7f). This result provides mechanistic evidence that Polγ deficiency-mediated autophagy increases cell proliferation via AKT activation.

## Discussion

In this study, we demonstrate for the first time that oxidative modification of Polγ and subsequent inactivation of Polγ selectively trigger prosurvival autophagy responses via Rictor-mediated mTORC2. mTOR is a key regulator of autophagy and cell growth [27]. Although the role of mTORC1 is well documented, the role of mTORC2 remains unclear. Here, we demonstrated that perturbation of mitochondrial energy metabolism due to deficiency of Polγ causes an increase in ROS, which activates Rictor to initiate prosurvival autophagy. Our findings support that activation of mTORC2 is a mitochondria-mediated event, at least under the conditions of mitochondrial oxidative stress due to Polγ deficiency.

It has been shown that nitric oxide and superoxide radical generated by UVB radiation can form peroxynitrite, leading to nitration of proteins [17, 18]. Specific tyrosine nitration and inactivation of enzymatic activity by peroxynitrite have been reported for MnSOD and aconitase [28, 29]. Our data indicate that Polγ is nitrated when human and mouse keratinocytes are exposed to UVB radiation, confirming our previous data for mouse skin tissues [9]. Importantly, we identified Y964 in the active site of DNA Polγ as the primary site of peroxynitrite-induced inactivation of Polγ (Fig. 1). Furthermore, tyrosine mutation to phenylalanine at position 964 of mouse Polγ suppressed the enzymatic activity, supporting the relevance of tyrosine nitration and loss of enzymatic function. Consistent with the function of Polγ in mtDNA repair, we observed significant mtDNA damage and impairment of mitochondrial function in Polγ-deficient cells that led to decreases in ATP-linked oxygen consumption, increased glycolysis, and higher ROS levels. We also have demonstrated that Polγ deficiency increases autophagy markers such as LC3 ll formation, beclin 1, and ATG7, as well as activates ribosomal kinase. These results are consistent with the concept that Polγ deficiency initiates signaling in prosurvival autophagy pathways.

The mechanism by which Polγ deficiency enhances prosurvival autophagy is unclear. Our data suggest that Polγ deficiency led to metabolic reprogramming in Polγ-deficient cells, which then generated ROS to activate mTOR pathways. It has been reported that the activation of mTOR is required in immune cells [30] during proliferation. Furthermore, endotoxin-induced autophagy is a survival mechanism that drives proliferation of hepatocytes [31] and in cardiac tissues [32]. The central components of the cell survival pathway, including AKT and mTOR, sense cellular metabolic alterations and trigger cell survival [24]. Our results demonstrate an increase in the phosphorylation of mTOR and AKT in Polγ-deficient cells (Fig. 5), supporting the previous findings that AKT-mTOR regulates autophagy and cell survival. Although it has been demonstrated that the PI3K-AKT-mTOR pathway mediates antiautophagic signaling, inhibition of mTOR by rapamycin induces autophagic cell death [33], and recent studies have shown that autophagy is also induced through activation of AKT-mTOR pathways [34, 35]. In yeast, the TOR complex-1, which is similar to the mTOR regulatory protein raptor that is a key regulator of translation and ribosome biogenesis, is responsible for the induction of autophagy [36]. Recently, it has been reported that AKT increases autophagy upon activation (by phosphorylation at S473) and consequently forms a complex with lysosomal proteins [37]. Serine-threonine kinase AKT and mTOR are well connected in their signaling pathway, whereas mTORC2 is known to phosphorylate AKT at S473. Our data show an increase in AKT phosphorylation at S473 as well as a preferential formation of mTORC2 over mTORC1 in Polγ-deficient cells (Fig. 5). Further, our strategy of overexpression and knockdown of Rictor, which is an important component of mTORC2, clearly demonstrates the role of Rictor in autophagy in Polγ-deficient cells (Fig. 6). Overexpression of Rictor mitigated the autophagic response in Rictor-deficient MEF cells, providing direct evidence that mTORC2 plays a critical role in inducing autophagic responses in Polγ-deficient cells.

**Fig. 8:**
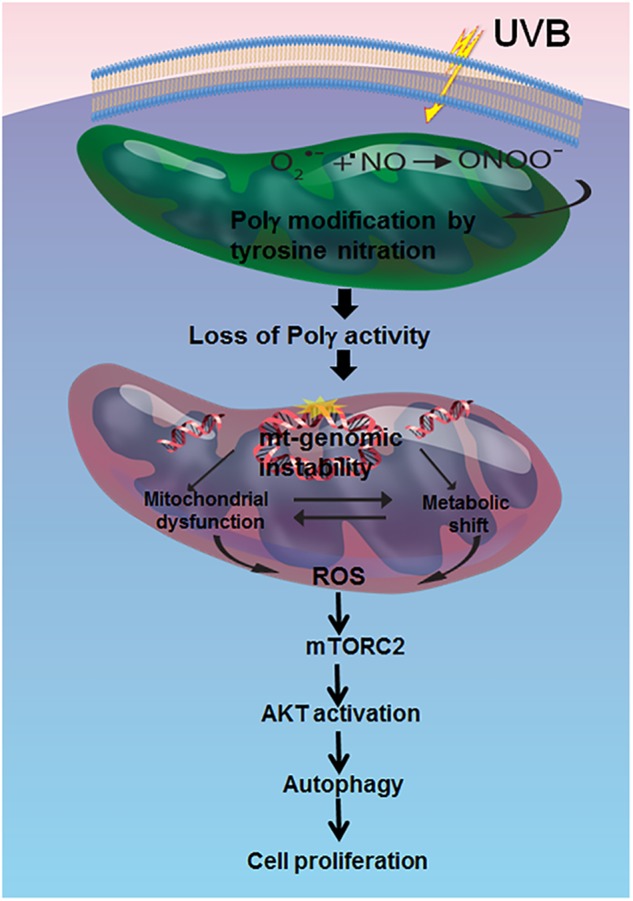
Summary of the proposed role of Polγ in prosurvival autophagy. Schematic diagram of the impact of Polγ deficiency and subsequent loss of enzymatic activity on the mTORC2/AKT pathway during prosurvival autophagy

Although the outcome of autophagosome formation and its consequences for cell survival depend on cell type and context [38, 39], autophagy clearly serves a critical role in cellular homeostasis. In this report, we demonstrate that nitration of Polγ and subsequent catalytic inactivation of Polγ trigger prosurvival autophagy responses selectively via the Rictor-mediated mTORC2, which is schematically depicted in Fig. 8. These results provide critical insights into the mechanism of mitochondrial dysfunction-mediated autophagy that have broad implications for our understanding of physiological and pathological conditions leading to the development of cancer and other metabolism-related diseases.

## Materials and methods

### Reagents

Anti-LC3A/B (Cat. 1274), anti-Beclin 1 (Cat. 3495), and ATG7 (Cat. 8558) were purchased from Cell Signaling Technology (Danvers, MA). Both anti-β-actin (Cat. SAB2100037), anti-mTOR (Cat. PLA0114), and anti-Flag (F3165) antibodies were purchased from Sigma (St. Louis, MO). The rabbit polyclonal Polγ (Cat. PA5-75881) was purchased from Thermo Scientific (Waltham, MA). Anti-Raptor (Cat. 05–1470) and anti-Rictor (Cat. 05–1471) antibodies were purchased from Millipore (Temecula, CA). Polγ siRNA (Cat. Sc-155884) and the lentiviral Polγ shRNA plasmid (Sc-155884-SH) were purchased from Santa Cruz Biotechnology (Santa Cruz, CA). PCR amplified full-length Polγ cDNA was sucloned into pEGFP-N1-Flag expression vector (Addgene, Cat. 60360), and the pEGFP-LC3 plasmid was a kind gift of Dr. Xianglin Shi, University of Kentucky (Lexington, KY). MnTnBuOE-2-PyP^5+^ (MnP) was the kind gift of Dr. Ines Batinic-Haberle, Duke University School of Medicine (Durham, NC). 3-Methyle adenine (3-MA, Cat. tlrl-3ma) and Bafilomycin A1 (Cat. tlrl-baf1) were purchased from InvivoGen (San Diego, CA). 10-nonylacridine orange bromide (NAO) was purchased from Thermo Fisher (Cat. A1372) All other chemicals were purchased from Sigma unless otherwise specified.

### Cell culture and treatments

The JB6 mouse skin epidermal cell line was originally obtained from Dr. Nancy H. Colburn of the National Cancer Institute, MD, and was maintained as described previously [40, 41]. The primary human epidermal keratinocyte (HEKn, Cat. C-001-5C) cells were purchased from Invitrogen Life Sciences (Carlsbad, CA). Both wild-type and Rictor-knockout MEFs were originally obtained from David Sabatini (Whitehead Institute, MA). All cells were grown in a 5% CO_2_ incubator at 37 °C in media consisting of either MEM supplemented with 10% fetal bovine serum (Hyclone Inc., Logan, UT), 1% (w/v) l-glutamine (Invitrogen), and 1% P/S antibiotics (Invitrogen) or Epilife medium supplemented with S7 (Invitrogen) for primary cells. Cells were exposed to a single dose of UV radiation (50 mJ/cm^2^ × 1 h) using UVB lamps as described previously [9]. Cells were also treated with 20 μM MnP, 2.5 mM 3-MA, and 50 nM Bafilomycin A1 for 24 h.

### Transfection

Cells were grown for 24 h with no antibiotics to obtain 70–80% confluency. The cells were then transfected with plasmids following a transfection protocol using Lipofectamine^Ⓡ^ as directed by the manufacturer. Cells were transfected with 1–2 μg of plasmid DNA containing either Polγ or LC3-GFP (equilibrated to the same amount of DNA by adding control vector) or control vector alone. Twenty-four hours after transfection, the cells were washed twice with phosphate-buffered saline and incubated in fresh medium for another 24 h with or without treatment for an indicated time. Cells were then processed for whole cell lysate preparation or fluorescence microscopy. Similarly, siRNAs were transfected using Transfectin^®^ (Santa Cruz Biotechnology) according to the manufacturer’s protocol. Cells were exposed to the siRNA for 72 h. The siRNA sequences targeting Polγ are presented in Supplementary Table S1.

### Polγ shRNA stable clones

Lentiviral constructs containing mouse Polγ shRNA (Santa Cruz Biotechnology) were used to make JB6 cells with stable knockdown of Polγ protein. Lentiviral constructs expressing an empty vector were used as controls. The lentiviral constructs were packaged using the manufacturer-provided trans-lentiviral packaging system before transduction into JB6 cells in accordance with the manufacturer’s protocol. For stably expressing Polγ shRNA, JB6 cells were selected using puromycin. Stable expression of Polγ in pools of cells was verified by western blotting using Polγ antibody.

### Deletion of the Polγ gene in JB6 cells by CRISPR

The D10A Cas9n CRISPR system [42] was used to introduce two DNA single-strand breaks to minimize off-target effects. The genome sequences containing the guide/pam sequences in the mouse Polγ gene are: (A) 5′-CCT/TCGTGGTAACCCTGTCCACT-3′ (reverse complementary) and (B) 5′-TTGCCTCTTTCAGGTATTCC/TGG-3′. The two sequences are adjacent, and thus by combination Cas9n introduces a DNA double-strand break in the beginning of exon 3 (for details, see Fig. 2 legend). To construct a Cas9n vector targeting site (A), a linker was prepared by annealing 5′-CACCGAGTGGACAGGGTTACCACGA-3′ and 5′-AAACTCGTGGTAACCCTGTCCACTC-3′, followed by phosphorylation of the 5′-ends by T4 polynucleotide kinase. The linker was then cloned into the pSpCas9n(BB)-AA-Puro vector (Addgene, Cambridge, MA; Cat. 62988) that had been prepared by cutting with BbsI. Similarly for (B), a linker (5′-CCGTTGCCTCTTTCAGGTATTCC-3′ annealed with 5′-AACGGAATACCTGAAAGAGGCAA-3′) was cloned into pD1411-AP (DNA2.0 Inc., Newark, CA) that had been cut with SapI. Deletion in the locus was confirmed by PCR of the genomic DNA using primers 5′-AAGACACAAGGGGTTGGTCC-3′ and 5′-AGACGGTGTTGTCAAAGTTTGT-3′, and then the absence of Polγ protein expression was confirmed by immunoblotting.

### In vitro kinase assay for mTORC1 and mTORC2 activity

The in vitro kinase assays for mTORC1 and mTORC2 activity were performed according to the protocol adopted from Dr. David Sabatini’s laboratory. Briefly, cells were lysed in 200 μL of lysis buffer containing 40 mm HEPES, pH 7.5, 120 mM NaCl, 0.3% CHAPS, 1 mM EDTA, 2.5 mM sodium pyrophosphate. Cell lysate was passed through a sepharose-affinity column conjugated with anti-raptor or anti-Rictor antibody (Cell Signaling, CA). After appropriate washing with a kinase buffer containing 1 mM dithiothreitol, beads were incubated for 30 min at 30 °C with purified recombinant S6K and AKT as substrate for mTORC1 and mTORC2 activity, respectively. The reactions were then terminated by boiling in the presence of 1× SDS sample buffer. Phosphorylation was detected by western blotting using anti-pp70S6K antibody for mTORC1 activity and antiphosphorylated AKT (S473) antibody for mTORC2 activity.

### Site-directed mutagenesis

Specific point mutation of Polγ was performed using the QuickChange Site-Directed Mutagenesis kit (Agilent Technologies, Santa Clara, CA; Cat. 200524) as described previously [43]. Primer sets were designed to mutate tyrosine (Y964) to phenylalanine in mouse Polγ using the primers (a) 5′-AGGCCTGCGCCGGCCTCGGCTGTCTGCG-3′ (forward) and

(b) 5′-CGCAGACAGCCGACCGGCGCAGGCCT-3′ (reverse). The clonal DNAs were propagated in *E. coli*. Clones carrying a single mutation were confirmed by DNA sequencing.

### Polγ reverse transcriptase activity assay for pure protein

The RNA-dependent DNA polymerase activity of purified Polγ was measured as described previously [44]. Briefly, a 50 μL reaction mixture with 10 μg of the mitochondrial protein in 25 mM HEPES-KOH pH 8.0, 0.5 mM MnCl_2_, 100 mM NaCl, and 2.5 mM β-mercaptoethanol; 50 μg/mL poly(rA); oligo(dT)_12–18_; 100 μg/mL acetylated bovine serum albumin (BSA); 0.1 mM aphidicolin; 500 μg/mL RNasin^®^ RNase inhibitor; and 5 μM [α-^32^P]thymidine 5′-triphosphate was incubated at 37 °C for 10 min. The reaction was stopped with 1.0 mL of stop solution (500 mM NaOH, 100 mM sodium pyrophosphate, 0.1 mg/mL calf thymus DNA, 0.5 mg/mL BSA). The DNA was precipitated with 20% trichloroacetic acid and radioactivity was measured by a liquid scintillation counter.

### Polγ activity assay in isolated mitochondria

Polγ activity in mitochondria was measured according to the method described by Szczesny [45]. Briefly, the activity of Polγ was measured using primer (5′-GACCCGATCTGATCCGATTCG-3′) and template (5′-ATCCAACCTCGCGGTCGTATCGAATCGGATCAGATCGGGTCGTCAA-3′). The primer−template was annealed and added to 20 μL of reaction mixture containing 50 mM Tris-HCl (pH 8.6), 50 mM KCl, 2 mM MgCl_2_, 20 μM each of three unlabeled dNTPs, 2 μCi of [α-^32^P] dATP and 3 μg of the mitochondria. After incubation at 37 °C for 30 min, the reaction was terminated by the addition of 5 μL of 70% formamide. The amplified DNA was resolved by 20% acrylamide/7 M urea gel electrophoresis. The radioactivity of the bands was quantified using Image Quanta software.

### Liquid chromatography electrospray ionization-tandem mass spectrometry (LC-ESI-MS/MS) analysis

The trypsin digestion and LC-MS/MS analysis of pure Polγ were performed as reported previously [42, 46]. The tryptic peptides were analyzed using an LTQ-Orbitrap mass spectrometer (Thermo Scientific, MA) coupled with an Eksigent Nanoflex cHiPLC™ system (Eksigent, CA) through a nano-electrospray ionization source. The peptides were separated with a reverse-phase cHiPLC at a flow rate of 300 nL/min. The LC-MS/MS data were submitted to a local mascot server for identification of peptide modifications via Proteome Discoverer (version 1.3) against the *musculus* taxonomy subset of the Swissprot database.

### Measurement of oxygen consumption rate and extracellular acidification rate

XF extracellular flux assays (Seahorse-Bioscience, MA) were utilized to measure the oxygen consumption rate (OCR) and the extracellular acidification rate (ECAR). The OCR experiments were performed by sequentially adding the substrate oligomycin, carbonyl cyanide-4-(trifluoromethoxy)phenylhydrazone (FCCP), and antimycin/rotenone to cells and the ECAR measurements were performed by using glucose, oligomycin, and 2-deoxyglucose as substrate.

### Western blotting

Proteins were analyzed by western blotting. Briefly, cell extracts were subjected to 10% or 12% SDS-polyacrylamide gel electrophoresis and transferred to a nitrocellulose membrane. Following blocking with 5% BSA, membranes were then probed with specific primary antibody (by diluting at a range from 500 to 5000) followed by secondary antibody (dilution range 2000 to 6000) to detect specific proteins. Protein bands were detected using the enhanced chemiluminescence detection system (ECL^®^, Amersham Bioscience). Densitometric analysis was performed for quantification of proteins using ImageJ software (NIH).

### Fluorescence microscopy

Green florescent LC3 puncta formation was detected by fluorescence microscope (model IX71, Tokyo, Japan) in live cells following transient transfection of pEGFP-LC3 expression vector.

### Immunoprecipitation

Immunoprecipitation studies were carried out as described previously [43] with whole cell extracts using specific antibodies in a binding buffer (9.1 mM NaHPO4, 1.7 mM NaH_2_PO_4_, 150 mM NaCl [pH 7.4], 0.1% Nonidet P-40, 0.5% sodium deoxycholate, 0.1% SDS) containing 10 μg/mL phenylmethyl sulfonyl fluoride and 1 μg/mL aprotinin as protease inhibitor. The antibodies used for immunoprecipitation were rabbit anti-3-nitrotyrosine, rabbit anti-mTOR, or rabbit anti-Rictor. One mg of protein from whole cell lysate was incubated overnight with 2 μg of corresponding antibody at 4 °C with continuous rotation, at which point 20 μL of protein A/G beads (Santa Cruz, CA) were added to the reaction mixture and the rotation continued for another 2 h at 4 °C. Immunoprecipitates were collected by centrifugation at 2500 × *g* for 5 min followed by washing with binding buffers. After the final wash, all the adhering liquids were removed from the beads. Samples were then suspended in 1× Laemmli buffer, subjected to SDS-polyacrylamide gel electrophoresis, and specific proteins were detected by western blotting.

### Quantification of ROS levels by flow cytometry

MitoSox Red (Thermo Scientific, Cat. M36008), a highly selective mitochondrial superoxide indicator for live cells, was used to measure ROS levels. Rotenone (200 nM, Sigma), known to be a mitochondrial superoxide inducer, was used as a positive control. To account for superoxide-specific fluorescence, the cells were pretreated with 100 units/mL PEG-SOD (Sigma) or 20 μM MnP for 24 h prior to measurement. In brief, cells were loaded with 5 μM MitoSox Red for 10 min at 37 °C and rinsed three times with HBSS and then collected by trypsinization. Cell suspension (final volume of 500 μL in 1× PBS) was analyzed using a BD FACS LSR II flow cytometer (Becton Dickinson). Ten thousand cells were acquired for each sample using the FACS DIVA software (Becton Dickinson). The results were then analyzed using Cell Quest Pro software.

### BrdU incorporation and detection assay

Cell proliferation was assessed based on 5-bromo-2′-deoxyuridine (BrdU) incorporation followed by ELISA according to the manufacturer’s protocol (Cell Biolabs, Inc.). Briefly, 30,000 cells were grown in a 96-well plate for 24 h at 37 °C in a 5% CO_2_ incubator. BrdU solutions were added to the well to achieve 1 nM final concentration and incubated for 6 h. Cells were then washed, fixed, and denatured at 37 °C for 30 min. Following successive washings with PBS, cellular BrdU uptake was detected by colorimetric ELISA (450 nm) using anti-BrdU antibody. Data were normalized to the protein concentration of the corresponding sample.

### Statistical analysis

Data were analyzed using one-way analysis of variance for multiple-group comparisons, and Student’s *t* test for two-group comparisons. For multiple-group statistics, Bonferroni’s post-test for multiple comparisons was used to determine the statistical significance.

## Electronic supplementary material

DNA polymerase gamma (Polγ) deficiency triggers a selective mTORC2 prosurvival autophagy response via mitochondria-mediated ROS signaling
