# Supplementary material for: DNA polymerase gamma (Polγ) deficiency triggers a selective mTORC2 prosurvival autophagy response via mitochondria-mediated ROS signaling
Source: Oncogene. 2018 Jul 23;37(48):6225–42. doi: 10.1038/s41388-018-0404-z (PMC6265263; doi:10.1038/s41388-018-0404-z)
Supplement: Supplementary file 1 — DNA polymerase gamma (Polγ) deficiency triggers a selective mTORC2 prosurvival autophagy response via mitochondria-mediated ROS signaling [file 41388_2018_404_MOESM1_ESM.docx]

**SUPPLEMENTAL INFORMATION**

**DNA polymerase gamma (Polγ) deficiency triggers a selective mTORC2 pro-survival autophagy response via mitochondria-mediated ROS signaling**

Sanjit K. Dhar^1^, Vasudevan Bakthavatchalu^2^, Bithika Dhar^1^, Jing Chen^3^, Izumi Tadahide^1^, Haining Zhu^3^, Tianyan Gao^3^, and Daret K. St. Clair^1^

^1^Department of Toxicology and Cancer Biology, University of Kentucky, Lexington, KY 40536 ^2^Division of Comparative Medicine, Massachusetts Institute of Technology, Cambridge, MA 02139, ^3^ Department of Molecular and Cellular Biochemistry, University of Kentucky, Lexington, KY 40536

Running Title: Deficiency of Polγ increases autophagy

Supplementary Table 1: Mouse Polγ si/shRNA sequences used to silence Polγ gene expression.

**DNA Polγ siRNA (m)**

Strand 1

Sense 5`- CGAUGAAGAAGUCGUUGUAtt-3`

Antisense 5`- UACAACGACUUCUUCAUCGtt-3`

Strand 2

Sense 5`- GGCAAACGGUAGAAGAACUtt-3`

Antisense 5`- AGUUCUUCUACCGUUUGCCtt-3`

Strand 3

Sense 5`- CUACAAGAUCUUCGAAUGAtt-3`

Antisense 5`- UCAUUCGAAGAUCUUGUAGtt-3`

**DNA Polγ shRNA (m)**

Strand 1

Sense 5`-GUGUAGAGUUACGAAAGAAtt-3`

Antisense 5`-UUCUUUCGUAACUCUACACtt-3`

Strand 2

Sense 5`-GAAAGUAUACGCGAAAUCUtt-3`

Antisense 5`-AGAUUUCGCGUAUACUUUCtt-3`

Strand 3

Sense 5`-GCUAUGAGUCCCUCUAACUtt-3`

Antisense 5`-AGUUAGAGGGACUCAUAGCtt-3`


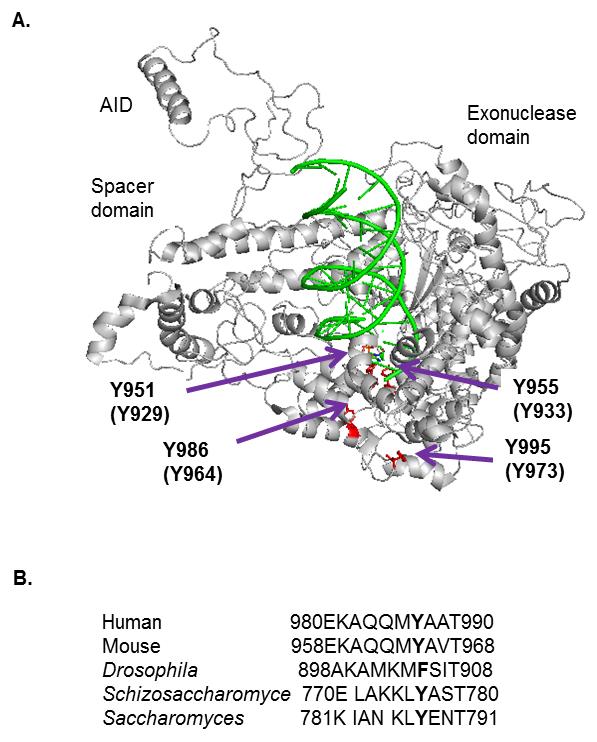


**Supplementary** **Figure S1.** (A) Structural model of human Polγ with DNA (green). The model was created by comparing with T7 DNA polymerase using PyMOL software (pymol.org). The locations of the tyrosine residues in the catalytic site of Polγ are indicated by arrows, and the amino acid side chains are displayed in red. Residue numbers are for the human protein, with the corresponding mouse protein residue numbers in parentheses. (B) Conservation of Y964 in mouse Polγ (986 in the human sequence) is shown in different species.


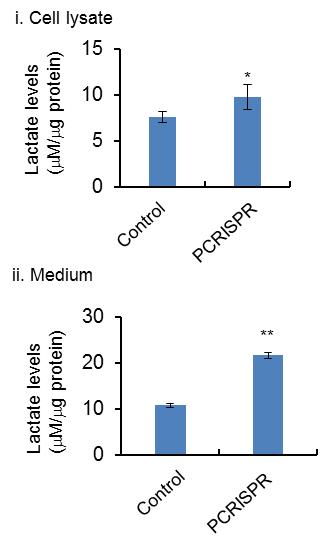


**Supplementary** **Figure S2.** Lactate concentration was measured using a lactate assay kit (Biomedical Research Service Center, University of Buffalo). The assay is based on the reduction of the tetrazolium salt INT in an NADH-coupled enzymatic reaction to formazan at an absorption maximum of 492 nm. (i) Cellular lactate levels and (ii) lactate levels in the medium were measured in Polγ-deficient PCRISPR and parental control cells (JB6). Each experiment was repeated at least three times. Each data point represents the mean ± SD of three individual samples and the statistical analysis was performed using t-tests. Statistical significance is indicated by asterisks: *p<0.05 and **p<0.01.

**Supplementary Figure S3.** (A) Effects of an AKT inhibitor (3-[1-[[4-(7-Phenyl-3H-imidazo[4,5-g]quinoxalin-6-yl)phenyl]methyl]piperidin-4-yl]-1H-benzimidazol-2-one) on Polγ deficiency-mediated autophagy. Polγ stable knockdown cells were treated with AKT inhibitor for 24 h and then western blotting was carried out using an anti-LC3 antibody to assess autophagy. p-AKT and LC3 II bands were densitometrically scanned and normalized with β-actin as the internal control. (B) Polγ deficiency-mediated activation of Rictor and AKT was determined in the absence or presence of mitochondrial antioxidant MnP. Each data point in the bar graphs represents the mean ± SD of three individual samples. C. Polγ-deficiency mediated increase of LC3 II formation was attenuated following suppression of AKT by siRNA. Suppression of AKT by siRNA overexpression was confirmed by western blotting. Statistical significance was determined by one-way ANOVA analysis and Bonferroni’s post-test for multiple-group comparisons. Statistical significance is indicated by asterisks: **p<0.01.

**Supplementary Figure S4.** (A) Effects of UVB radiation on autophagy. LC3 punctation was detected in cells treated with UVB radiation. For each cell type, 100 GFP-positive cells were counted, and the bottom panel shows the quantification of punctated cells. (B) JB6 cells are treated with UVB radiation and then the LC3 II formation is detected by western blotting using LC3 antibody following overexpression of wild-type or mutant Polγ expression vector. The band intensity of LC3 II is quantified and normalized with β-actin, as loading control. The overexpression of ectopically expressed Polγ protein was confirmed by western blotting using Flag antibody. . In the bar graphs, each data point represents the mean ± SD of three individual samples. Statistical analysis was performed using one-way ANOVA analysis and Bonferroni’s post-test for multiple-group comparisons. Statistical significance is indicated by asterisks: *p<0.05 and **p<0.01.

**Supplementary Figure S5.** Characterization of PCRISPR cells. Polγ protein expression was diminished in PCRISPR cells as compared to parental control cells were detected by western blotting. Overexpression of wild-type and mutant Polγ proteins was confirmed by western blotting using Flag or Polγ antibody. The protein levels of mitochondrially expressed MnSOD were detected by western blotting using MnSOD antibody. Effect of Polγ suppression and overexpression in PCRISPR cells on autophagy was detected by measuring LC3 II formation. . Each data point represents the mean ± SD of three individual samples. Statistical analysis was performed using one-way ANOVA analysis and Bonferroni’s post-test for multiple-group comparisons. Statistical significance is indicated by asterisks: *p<0.05 and **p<0.01.
